# Supplementary material for: Hot spot prediction in protein-protein interactions by an ensemble system
Source: BMC Syst Biol. 2018 Dec 31;12(Suppl 9):132. doi: 10.1186/s12918-018-0665-8 (PMC6311905; doi:10.1186/s12918-018-0665-8)
Supplement: Supplementary file 2 — All relevant MATLAB code. A simple Matlab implement of our predictor. (ZIP 19,742 kb) [file 12918_2018_665_MOESM2_ESM.zip › Additional file 2/Read ASA/ASA Property/BID/1DZIA.txt]

 NetSurfP - Protein Surface Accessibility and Secondary Structure Predictions

  

|  |  |  |
| --- | --- | --- |
|  |  | NetSurfP - Protein Surface Accessibility and Secondary Structure Predictions  Technical University of Denmark |

  


---

```
# For publication of results, please cite:
# A generic method for assignment of reliability scores applied to solvent accessibility predictions.
# Bent Petersen, Thomas Nordahl Petersen, Pernille Andersen, Morten Nielsen and Claus Lundegaard
# BMC Structural Biology 2009, 9:51 doi:10.1186/1472-6807-9-51
#
# Column 1: Class assignment - B for buried or E for Exposed - Threshold: 25% exposure, but not based on RSA
# Column 2: Amino acid
# Column 3: Sequence name
# Column 4: Amino acid number
# Column 5: Relative Surface Accessibility - RSA
# Column 6: Absolute Surface Accessibility
# Column 7: Z-fit score for RSA prediction
# Column 8: Probability for Alpha-Helix
# Column 9: Probability for Beta-strand
# Column 10: Probability for Coil
E A  1DZIA                  1    0.624  68.765  -0.058   0.003   0.003   0.994
E L  1DZIA                  2    0.373  68.296   0.710   0.019   0.141   0.840
B I  1DZIA                  3    0.086  15.928   0.178   0.004   0.616   0.381
B D  1DZIA                  4    0.179  25.722   0.547   0.002   0.816   0.182
B V  1DZIA                  5    0.033   5.087   0.512   0.001   0.959   0.040
B V  1DZIA                  6    0.041   6.379   0.500   0.000   0.983   0.017
B V  1DZIA                  7    0.024   3.750   1.434   0.000   0.983   0.017
B V  1DZIA                  8    0.019   2.905   1.461   0.000   0.983   0.017
B C  1DZIA                  9    0.026   3.622   1.128   0.001   0.900   0.099
B D  1DZIA                 10    0.071  10.217  -0.142   0.004   0.616   0.381
B E  1DZIA                 11    0.224  39.203  -0.353   0.019   0.141   0.840
B S  1DZIA                 12    0.086  10.114  -1.100   0.018   0.047   0.935
E N  1DZIA                 13    0.417  61.122  -0.791   0.018   0.019   0.964
E S  1DZIA                 14    0.271  31.714  -0.879   0.018   0.019   0.964
B I  1DZIA                 15    0.153  28.342  -0.578   0.058   0.017   0.925
E Y  1DZIA                 16    0.492 105.119  -0.768   0.016   0.005   0.979
E P  1DZIA                 17    0.471  66.877   0.397   0.406   0.004   0.590
B W  1DZIA                 18    0.188  45.310   0.146   0.923   0.002   0.076
E D  1DZIA                 19    0.620  89.400   0.262   0.970   0.001   0.030
E A  1DZIA                 20    0.327  36.090   1.658   0.970   0.001   0.030
B V  1DZIA                 21    0.026   3.981   0.650   0.970   0.001   0.030
B K  1DZIA                 22    0.184  37.890   1.649   0.970   0.001   0.030
E N  1DZIA                 23    0.506  74.078   1.767   0.970   0.001   0.030
B F  1DZIA                 24    0.049   9.814   0.936   0.988   0.000   0.012
B L  1DZIA                 25    0.018   3.314   1.170   0.988   0.000   0.012
E E  1DZIA                 26    0.303  53.004   1.777   0.988   0.000   0.012
E K  1DZIA                 27    0.369  75.986   2.097   0.988   0.000   0.012
B F  1DZIA                 28    0.041   8.329   0.450   0.970   0.001   0.030
B V  1DZIA                 29    0.054   8.361   0.054   0.970   0.001   0.030
E Q  1DZIA                 30    0.543  97.033   1.249   0.923   0.002   0.076
E G  1DZIA                 31    0.363  28.529   1.162   0.858   0.002   0.139
B L  1DZIA                 32    0.054   9.961   0.634   0.694   0.003   0.303
E D  1DZIA                 33    0.489  70.450   0.200   0.502   0.002   0.495
E I  1DZIA                 34    0.477  88.171  -1.156   0.257   0.016   0.727
E G  1DZIA                 35    0.295  23.224  -0.805   0.058   0.017   0.925
E P  1DZIA                 36    0.485  68.892  -0.560   0.018   0.019   0.964
E T  1DZIA                 37    0.669  92.776  -0.406   0.018   0.019   0.964
E K  1DZIA                 38    0.383  78.824  -0.092   0.005   0.015   0.979
B T  1DZIA                 39    0.056   7.739  -0.231   0.004   0.197   0.799
B Q  1DZIA                 40    0.104  18.503   0.295   0.002   0.816   0.182
B V  1DZIA                 41    0.026   4.042   0.445   0.001   0.959   0.040
B G  1DZIA                 42    0.022   1.708   0.578   0.001   0.959   0.040
B L  1DZIA                 43    0.019   3.479   1.165   0.001   0.959   0.040
B I  1DZIA                 44    0.026   4.847   0.692   0.001   0.959   0.040
B Q  1DZIA                 45    0.093  16.538   0.369   0.001   0.959   0.040
B Y  1DZIA                 46    0.050  10.706   0.396   0.001   0.900   0.099
B A  1DZIA                 47    0.163  17.963  -0.963   0.005   0.262   0.733
E N  1DZIA                 48    0.457  66.876  -1.101   0.005   0.045   0.951
E N  1DZIA                 49    0.420  61.532   0.044   0.005   0.336   0.660
B P  1DZIA                 50    0.078  10.997  -0.570   0.004   0.514   0.481
E R  1DZIA                 51    0.290  66.387   0.328   0.018   0.846   0.136
B V  1DZIA                 52    0.252  38.809  -0.276   0.001   0.900   0.099
B V  1DZIA                 53    0.233  35.797  -0.634   0.011   0.918   0.071
B F  1DZIA                 54    0.073  14.751   0.396   0.003   0.718   0.279
E N  1DZIA                 55    0.365  53.465  -0.098   0.004   0.420   0.576
B L  1DZIA                 56    0.088  16.113  -1.145   0.004   0.085   0.910
E N  1DZIA                 57    0.382  55.939  -0.567   0.005   0.015   0.979
E T  1DZIA                 58    0.495  68.670  -0.333   0.018   0.019   0.964
E Y  1DZIA                 59    0.330  70.457  -1.217   0.257   0.016   0.727
E K  1DZIA                 60    0.478  98.407  -1.087   0.257   0.016   0.727
E T  1DZIA                 61    0.429  59.558  -0.593   0.181   0.016   0.803
E K  1DZIA                 62    0.295  60.661   0.607   0.970   0.001   0.030
E E  1DZIA                 63    0.579 101.221   0.148   0.970   0.001   0.030
E E  1DZIA                 64    0.408  71.347   1.435   0.970   0.001   0.030
B M  1DZIA                 65    0.050  10.065   0.496   0.970   0.001   0.030
B I  1DZIA                 66    0.235  43.512   0.634   0.970   0.001   0.030
E V  1DZIA                 67    0.479  73.638   1.254   0.970   0.001   0.030
B A  1DZIA                 68    0.170  18.745   0.005   0.923   0.002   0.076
B T  1DZIA                 69    0.079  10.971  -1.642   0.858   0.002   0.139
E S  1DZIA                 70    0.485  56.865  -0.102   0.802   0.014   0.185
E Q  1DZIA                 71    0.517  92.390   0.091   0.622   0.015   0.363
B T  1DZIA                 72    0.095  13.107   0.127   0.430   0.016   0.555
E S  1DZIA                 73    0.467  54.732   0.142   0.278   0.093   0.628
E Q  1DZIA                 74    0.380  67.814  -1.341   0.191   0.086   0.723
E Y  1DZIA                 75    0.282  60.306  -0.861   0.113   0.043   0.844
E G  1DZIA                 76    0.444  34.919  -2.140   0.058   0.017   0.925
E G  1DZIA                 77    0.471  37.052  -2.628   0.058   0.017   0.925
E D  1DZIA                 78    0.416  59.989  -1.759   0.058   0.017   0.925
B L  1DZIA                 79    0.227  41.564  -1.459   0.115   0.016   0.868
B T  1DZIA                 80    0.051   7.032  -2.015   0.113   0.043   0.844
B N  1DZIA                 81    0.142  20.774  -0.570   0.354   0.048   0.598
B T  1DZIA                 82    0.048   6.602  -0.326   0.802   0.014   0.185
B F  1DZIA                 83    0.110  22.157  -1.454   0.923   0.002   0.076
E G  1DZIA                 84    0.282  22.233   0.926   0.970   0.001   0.030
B A  1DZIA                 85    0.018   2.028  -0.254   0.970   0.001   0.030
B I  1DZIA                 86    0.026   4.773   0.411   0.970   0.001   0.030
E Q  1DZIA                 87    0.323  57.670   1.262   0.970   0.001   0.030
E Y  1DZIA                 88    0.246  52.570   1.042   0.970   0.001   0.030
B A  1DZIA                 89    0.029   3.240  -0.495   0.970   0.001   0.030
B R  1DZIA                 90    0.093  21.366   0.507   0.970   0.001   0.030
E K  1DZIA                 91    0.509 104.640   1.288   0.970   0.001   0.030
E Y  1DZIA                 92    0.244  52.143   0.895   0.970   0.001   0.030
B A  1DZIA                 93    0.043   4.695  -0.934   0.923   0.002   0.076
E Y  1DZIA                 94    0.290  61.973  -0.064   0.858   0.002   0.139
E S  1DZIA                 95    0.559  65.538   0.169   0.782   0.003   0.216
E A  1DZIA                 96    0.476  52.455  -0.960   0.600   0.003   0.397
E A  1DZIA                 97    0.504  55.541  -1.159   0.502   0.002   0.495
E S  1DZIA                 98    0.503  58.952  -1.079   0.430   0.016   0.555
E G  1DZIA                 99    0.506  39.791  -1.492   0.257   0.016   0.727
E G  1DZIA                100    0.419  32.960  -1.899   0.115   0.016   0.868
B R  1DZIA                101    0.266  60.937  -0.492   0.115   0.016   0.868
E R  1DZIA                102    0.653 149.606  -1.047   0.115   0.016   0.868
E S  1DZIA                103    0.589  69.078  -0.819   0.058   0.017   0.925
B A  1DZIA                104    0.197  21.709  -0.676   0.018   0.019   0.964
E T  1DZIA                105    0.316  43.788   0.539   0.004   0.085   0.910
B K  1DZIA                106    0.198  40.831   1.405   0.004   0.514   0.481
B V  1DZIA                107    0.035   5.395   0.530   0.001   0.900   0.099
B M  1DZIA                108    0.013   2.501   1.789   0.001   0.959   0.040
B V  1DZIA                109    0.015   2.352   1.707   0.000   0.983   0.017
B V  1DZIA                110    0.013   1.937   1.728   0.001   0.959   0.040
B V  1DZIA                111    0.019   2.951   1.257   0.001   0.900   0.099
B T  1DZIA                112    0.029   4.036   0.053   0.003   0.718   0.279
B D  1DZIA                113    0.100  14.453  -0.038   0.004   0.138   0.858
B G  1DZIA                114    0.098   7.697  -0.765   0.018   0.019   0.964
E E  1DZIA                115    0.420  73.322   0.773   0.018   0.019   0.964
E S  1DZIA                116    0.346  40.598  -1.202   0.018   0.019   0.964
E H  1DZIA                117    0.457  83.110  -1.913   0.016   0.005   0.979
E D  1DZIA                118    0.509  73.289  -1.658   0.016   0.005   0.979
E G  1DZIA                119    0.579  45.528  -1.841   0.053   0.005   0.942
E S  1DZIA                120    0.458  53.701  -1.503   0.246   0.004   0.750
E M  1DZIA                121    0.469  93.787   0.199   0.176   0.004   0.820
B L  1DZIA                122    0.079  14.410   0.191   0.858   0.002   0.139
E K  1DZIA                123    0.359  73.764   0.820   0.923   0.002   0.076
E A  1DZIA                124    0.537  59.144   1.556   0.970   0.001   0.030
B V  1DZIA                125    0.055   8.469   0.287   0.970   0.001   0.030
B I  1DZIA                126    0.026   4.791   0.131   0.970   0.001   0.030
E D  1DZIA                127    0.516  74.341   1.929   0.970   0.001   0.030
E Q  1DZIA                128    0.383  68.404   2.011   0.970   0.001   0.030
B C  1DZIA                129    0.040   5.602  -0.536   0.923   0.002   0.076
E N  1DZIA                130    0.421  61.664   1.121   0.858   0.002   0.139
E H  1DZIA                131    0.769 139.881   0.816   0.600   0.003   0.397
E D  1DZIA                132    0.489  70.523   1.032   0.053   0.005   0.942
E N  1DZIA                133    0.679  99.464   0.371   0.005   0.015   0.979
B I  1DZIA                134    0.034   6.346   0.434   0.015   0.102   0.884
E L  1DZIA                135    0.313  57.329   2.090   0.001   0.900   0.099
B R  1DZIA                136    0.036   8.175   0.723   0.000   0.983   0.017
B F  1DZIA                137    0.040   8.128   1.063   0.000   0.983   0.017
B G  1DZIA                138    0.018   1.432   1.057   0.000   0.983   0.017
B I  1DZIA                139    0.024   4.385   0.869   0.001   0.959   0.040
B A  1DZIA                140    0.025   2.744  -0.742   0.001   0.900   0.099
B V  1DZIA                141    0.040   6.148   0.019   0.004   0.514   0.481
B L  1DZIA                142    0.075  13.733  -1.046   0.004   0.138   0.858
E G  1DZIA                143    0.321  25.271  -0.956   0.018   0.047   0.935
E Y  1DZIA                144    0.369  78.941  -1.584   0.058   0.017   0.925
B L  1DZIA                145    0.296  54.161  -1.259   0.115   0.016   0.868
E N  1DZIA                146    0.350  51.255  -1.068   0.176   0.004   0.820
E R  1DZIA                147    0.463 106.073  -1.071   0.321   0.003   0.675
E N  1DZIA                148    0.551  80.637  -1.168   0.321   0.003   0.675
B A  1DZIA                149    0.264  29.137  -0.854   0.321   0.003   0.675
B L  1DZIA                150    0.231  42.259  -1.564   0.339   0.016   0.645
E D  1DZIA                151    0.513  73.894  -0.877   0.257   0.016   0.727
E T  1DZIA                152    0.456  63.303  -0.426   0.339   0.016   0.645
E K  1DZIA                153    0.446  91.824  -1.139   0.522   0.016   0.462
B N  1DZIA                154    0.302  44.198  -1.022   0.430   0.016   0.555
E L  1DZIA                155    0.323  59.196  -0.166   0.430   0.016   0.555
E I  1DZIA                156    0.296  54.834   0.586   0.923   0.002   0.076
E K  1DZIA                157    0.590 121.425   0.592   0.970   0.001   0.030
E E  1DZIA                158    0.260  45.370   1.268   0.970   0.001   0.030
B I  1DZIA                159    0.026   4.865   0.432   0.970   0.001   0.030
E K  1DZIA                160    0.396  81.457   1.736   0.970   0.001   0.030
E A  1DZIA                161    0.513  56.489   1.653   0.923   0.002   0.076
B I  1DZIA                162    0.047   8.695   0.462   0.858   0.002   0.139
B A  1DZIA                163    0.048   5.279  -1.019   0.717   0.014   0.269
E S  1DZIA                164    0.585  68.585   0.030   0.257   0.016   0.727
E I  1DZIA                165    0.473  87.468   0.526   0.115   0.016   0.868
E P  1DZIA                166    0.528  74.923  -0.754   0.053   0.005   0.942
E T  1DZIA                167    0.644  89.267  -1.653   0.016   0.005   0.979
E E  1DZIA                168    0.487  85.131  -0.898   0.018   0.019   0.964
E R  1DZIA                169    0.540 123.637   0.132   0.004   0.085   0.910
B Y  1DZIA                170    0.153  32.589   0.791   0.003   0.718   0.279
B F  1DZIA                171    0.134  26.834   0.266   0.001   0.900   0.099
B F  1DZIA                172    0.067  13.427   0.717   0.001   0.900   0.099
E N  1DZIA                173    0.305  44.667   1.061   0.002   0.816   0.182
B V  1DZIA                174    0.036   5.533  -0.550   0.004   0.514   0.481
E S  1DZIA                175    0.616  72.183   0.685   0.018   0.047   0.935
E D  1DZIA                176    0.362  52.164   0.696   0.058   0.017   0.925
B E  1DZIA                177    0.211  36.879   0.154   0.782   0.003   0.216
E A  1DZIA                178    0.691  76.137   1.176   0.858   0.002   0.139
E A  1DZIA                179    0.380  41.865   1.798   0.858   0.002   0.139
B L  1DZIA                180    0.044   7.983   0.222   0.923   0.002   0.076
E L  1DZIA                181    0.463  84.684   1.946   0.858   0.002   0.139
E E  1DZIA                182    0.728 127.094   1.759   0.858   0.002   0.139
B K  1DZIA                183    0.184  37.911  -0.083   0.782   0.003   0.216
E A  1DZIA                184    0.333  36.653  -0.219   0.522   0.016   0.462
E G  1DZIA                185    0.886  69.736   0.047   0.003   0.003   0.994

Explain the output.  Go back.
```

---
